# Production and separation of ^43^Sc for radiopharmaceutical purposes

**DOI:** 10.1186/s41181-017-0033-9

**Published:** 2017-11-25

**Authors:** Katharina A. Domnanich, Robert Eichler, Cristina Müller, Sara Jordi, Vera Yakusheva, Saverio Braccini, Martin Behe, Roger Schibli, Andreas Türler, Nicholas P. van der Meulen

**Affiliations:** 1 Laboratory of Radiochemistry, Paul Scherrer Institute, 5232 Villigen PSI, Switzerland; 2 Department of Chemistry and Biochemistry, University of Bern, 3012 Bern, Switzerland; 3 Center for Radiopharmaceutical Sciences ETH-PSI-USZ, Paul Scherrer Institute, 5232 Villigen PSI, Switzerland; 4 Department of Chemistry and Applied Biosciences, ETH Zurich, 8093 Zurich, Switzerland; 5 GSI Helmholtzzentrum für Schwerionenforschung GmbH, 64291 Darmstadt, Germany; 6 Albert Einstein Center for Fundamental Physics (AEC), Laboratory for High Energy Physics (LHEP), University of Bern, 3012 Bern, Switzerland

**Keywords:** Radionuclide production, Cyclotron, ^43^Sc, ^43^Ca, ^46^Ti, Radiolabeling, PET phantom, PET/CT imaging

## Abstract

**Background:**

The favorable decay properties of ^43^Sc and ^44^Sc for PET make them promising candidates for future applications in nuclear medicine. An advantage ^43^Sc (T_1/2_ = 3.89 h, Eβ^+^_av_ = 476 keV [88%]) exhibits over ^44^Sc, however, is the absence of co-emitted high energy γ-rays. While the production and application of ^44^Sc has been comprehensively discussed, research concerning ^43^Sc is still in its infancy. This study aimed at developing two different production routes for ^43^Sc, based on proton irradiation of enriched ^46^Ti and ^43^Ca target material.

**Results:**

^43^Sc was produced *via* the ^46^Ti(p,α)^43^Sc and ^43^Ca(p,n)^43^Sc nuclear reactions, yielding activities of up to 225 MBq and 480 MBq, respectively. ^43^Sc was chemically separated from enriched metallic ^46^Ti (97.0%) and ^43^CaCO_3_ (57.9%) targets, using extraction chromatography. In both cases, ~90% of the final activity was eluted in a small volume of 700 μL, thereby, making it suitable for direct radiolabeling. The prepared products were of high radionuclidic purity, i.e. 98.2% ^43^Sc were achieved from the irradiation of ^46^Ti, whereas the product isolated from irradiated ^43^Ca consisted of 66.2% ^43^Sc and 33.3% ^44^Sc. A PET phantom study performed with ^43^Sc, *via* both nuclear reactions, revealed slightly improved resolution over ^44^Sc. In order to assess the chemical purity of the separated ^43^Sc, radiolabeling experiments were performed with DOTANOC, attaining specific activities of 5–8 MBq/nmol, respectively, with a radiochemical yield of >96%.

**Conclusions:**

It was determined that higher ^43^Sc activities were accessible *via* the ^43^Ca production route, with a comparatively less complex target preparation and separation procedure. The product isolated from irradiated ^46^Ti, however, revealed purer ^43^Sc with minor radionuclidic impurities. Based on the results obtained herein, the ^43^Ca route features some advantages (such as higher yields and direct usage of the purchased target material) over the ^46^Ti path when aiming at ^43^Sc production on a routine basis.

**Electronic supplementary material:**

The online version of this article (10.1186/s41181-017-0033-9) contains supplementary material, which is available to authorized users.

## Background

Nuclear imaging methods offer the possibility to follow disease processes in the body on a cellular and molecular level, thus, providing valuable information to oncology, cardiology and neurology (Bybel et al. 2008, Kitson et al. 2009). The two most widely-employed imaging techniques in nuclear medicine are Single Photon Emission Computed Tomography (SPECT) and Positron Emission Tomography (PET) (Ramogida and Orvig 2013). Traditionally, short-lived, non-metallic PET radionuclides such as ^11^C, ^13^N, ^15^O and, primarily, ^18^F are used as tracers by their incorporation into small organic molecules via covalent bonds. However, radiolabeling of peptide, antibody and other protein-based targeting agents is hampered by elaborated radiosynthetic processes necessary to introduce short-lived radionuclides into more complex molecular structures (Wadas et al. 2010). Metallic radionuclides usually feature prolonged decay times and, thus, they are considered to be better matches for the previously-mentioned targeting moieties, having long biological half-lives. The incorporation of such radiometals into a chelator, which itself is conjugated to a biomolecule, becomes possible by exploiting their vast coordination chemistry (Wadas et al. 2010, Ramogida and Orvig 2013).

The radiometal ^68^Ga achieved an important role in oncological PET (Banerjee and Pomper 2013, Velikyan 2014), as its decay characteristics (T_1/2_ = 68 min, Eβ^+^_av_ = 830 keV, [89%]) allow the acquisition of high quality images, for example, the visualization of neuroendocrine tumors and their metastases by ^68^Ga-labeled somatostatin analogues, as demonstrated in a number of clinical studies (Gabriel et al. 2007, Kwekkeboom et al. 2010). The commercial ^68^Ge/^68^Ga generator system ensures an easy and flexible availability of ^68^Ga, however, only a limited quantity of radioactivity (equivalent to two patient doses when using a new generator) can be obtained per elution (Eppard et al. 2013, Rösch 2013). The short half-life of ^68^Ga entails a close proximity of the production facility in question, which is obliged to follow the guidelines of good manufacturing practice (GMP) in most countries, to an operating PET scanner (Breeman, et al. 2011). The feasibility of centralizing the production and distribution of ^68^Ga-radiopharmaceuticals is compromised by the resulting high overall costs which, in turn, encouraged the quest for alternate options.

In this respect, ^44^Sc was proposed as a suitable alternative to ^68^Ga for clinical PET imaging (Pruszynski et al. 2010, Rösch 2012). Its decay is characterized by the emission of positrons with lower energy (Eβ^+^_av_ = 632 keV [94%]) compared to ^68^Ga, allowing for PET imaging with a potentially improved spatial resolution (Bunka et al. 2016, Domnanich et al. 2016). Considering its physical half-life of 4.04 h (Garcia-Torano et al. 2016), centralized production of radiopharmaceuticals and their transportation to remotely-located hospitals becomes attainable. Additionally, with the employment of ^44^Sc, radiolabeling of a broader variety of biomolecules with slower pharmacokinetic profiles comes within reach (Chakravarty et al. 2014, van der Meulen et al. 2015). The production of ^44^Sc in sufficient amounts for radiopharmaceutical purposes, as well as the in vitro and in vivo characterizations of ^44^Sc-labeled compounds, was the topic of a number of studies (Rösch and Baum 2011, Pruszynski et al. 2012, Müller et al. 2013, Chakravarty et al. 2014, Hernandez et al. 2014, Singh et al. 2017). Moreover, the chemical behavior of Sc(III) was shown to more closely resemble those of the other rare earth elements, which are commonly used as therapeutics (e.g., ^90^Y and ^177^Lu) (Reubi et al. 2000, Majkowska-Pilip and Bilewicz 2011, Müller et al. 2013, Umbricht et al. 2017). It is hypothesized, however, that the clinical application of ^44^Sc may be compromised by the high dose burden to the personnel caused by the co-emission of 1157 keV γ-rays with 99.9% intensity.

**Fig. 1:**
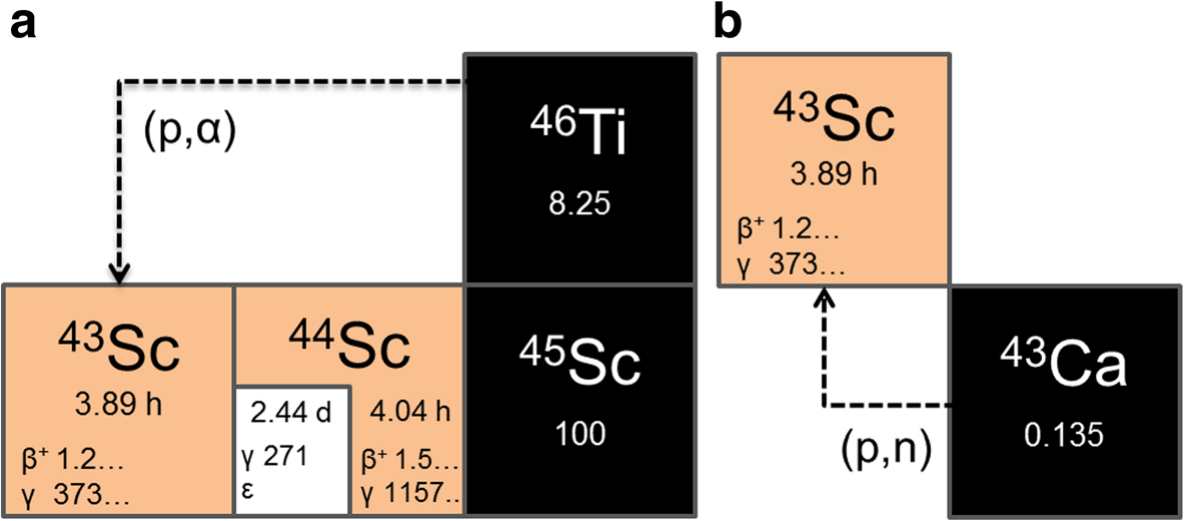
Production of ^43^Sc from ^46^Ti (**a**) and ^43^Ca (**b**) via the nuclear reactions ^46^Ti(p,α)^43^Sc and ^43^Ca(p,n)^43^Sc, respectively

It was since proposed to introduce another positron-emitting scandium radionuclide – ^43^Sc – which encompasses similarly favorable decay characteristics as ^44^Sc, but comes with a main γ-line of much lower energy and intensity (T_1/2_ = 3.89 h, Eβ^+^_av_ = 476 keV [88%], Eγ = 372 keV [23%]) (Walczak et al. 2015). To date, successful production of ^43^Sc was described by α-particle irradiation of natural calcium and enriched ^40^Ca through the nuclear reactions ^40^Ca(α,p)^43^Sc and ^40^Ca(α,n)^43^Ti → ^43^Sc, respectively. The obtained product was of high radionuclidic purity, and after its separation from the target material, successful radiolabeling was demonstrated using a derivative of the macrocyclic polyaminocarboxylic chelator 1,4,7,10-tetraazacyclododecane-1,4,7,10-tetraacetic acid (DOTA) (Szkliniarz et al. 2015, Walczak et al. 2015, Szkliniarz et al. 2016). Deuteron irradiation of enriched ^42^Ca targets was suggested as another possible ^43^Sc production channel, via the ^42^Ca(d,n)^43^Sc nuclear reaction (Walczak et al. 2015), however, the number of cyclotrons providing α-particle or deuteron beams is limited. The nuclear reactions ^43^Ca(p,n)^43^Sc and ^46^Ti(p,α)^43^Sc (Fig. 1) require only protons with low energies (<50 MeV) (International Atomic Energy Agency 2008, Koning et al. 2015, Experimental Nuclear Reaction Data (EXFOR) 2017), which can be generated by most biomedical cyclotrons and renders large-scale ^43^Sc production possible.

In this work, the feasibility of proton-induced production of ^43^Sc using ^43^Ca and ^46^Ti as target materials has been demonstrated – to our knowledge – for the first time, with the final product quality tested by means of radiolabeling. The image resolution of ^43^Sc was investigated and compared to that of ^44^Sc using Derenzo phantoms and a preclinical PET scanner.

## Methods

### Chemicals

Enriched ^46^TiO_2_ (97.0 ± 0.2% ^46^Ti, 0.44% ^47^Ti, 2.28% ^48^Ti, 0.15% ^49^Ti, 0.13% ^50^Ti, Isoflex, USA) was reduced to metallic ^46^Ti powder with calcium hydride (CaH_2_, 98% metals basis, Mg <1%, Alfa Aesar, Germany; 99.9% trace metals basis, Sigma Aldrich, USA), argon (Ar, 99.9999%, Linde, Germany) and acetic acid (CH_3_COOH, 100% Suprapur, Merck, Germany) and then used as target material. Prior to irradiation, a preceding scan for trace metals (Ca, Cr, Cu, Fe, Ir, K, Mg, Mn, Mo, Na, Sb, Si, Sn, Sr, Ti, U, Y, Zn, Zr) was performed by ICP-OES (Perkin Elmer Optima 3000). Enriched ^43^CaCO_3_ (28.5% ^40^Ca, 1.05% ^42^Ca, 57.9 ± 1.8% ^43^Ca, 12.36% ^44^Ca, <0.003% ^46^Ca, 0.19% ^48^Ca, Trace Sciences International, USA) and graphite powder (99.9999%, Alfa Aesar, Germany) were used for the preparation of ^43^Ca targets. The chemical separation of Sc(III) from Ti(III) and Ca(II) was performed with *N,N,N′,N′*-tetra-n-octyldiglycolamide, non-branched resin (DGA, particle size 50–100 μm, TrisKem International, France). SCX cation exchange cartridges (100 mg Bond Elut SCX, particle size 40 μm, Agilent Technologies Inc., USA) served for the concentration of Sc(III). Furthermore, MilliQ water, hydrochloric acid (HCl, 30% Suprapur, Merck, Germany) and sodium chloride (NaCl, Trace Select, ≥99.999%, Fluka Analytical, Germany) were used for the chemical separation procedures. The application of oxalic acid dihydrate ((COOH)_2_·2H_2_O, Trace Select, ≥99.9999% metals basis, Fluka Analytical, Germany) and ammonia solution (NH_3_, 25%, Suprapur, Merck, Germany) enabled full recycling of the target material. DOTANOC acetate was obtained from ABX GmbH, Germany, and used for the radiolabeling of the final product as a means of quality control.

### Reduction of enriched ^46^TiO_2_

The reduction of ^46^TiO_2_ to metallic ^46^Ti was performed with calcium hydride (Alfa Aesar) at Helmholtzzentrum für Schwerionenforschung (GSI) in Darmstadt, Germany. The detailed procedure has been outlined elsewhere (Lommel et al. 2014).

In order to increase the reduction yield, the reduction process for ^46^TiO_2_ was optimized at the Paul Scherrer Institute (PSI) with natural TiO_2_. Enriched ^46^TiO_2_ and ^nat^TiO_2_ (1.15 × 10^−3^ mol per tablet), respectively, were mixed with a 2–4 fold molar excess of CaH_2_ (2.3–4.6 × 10^−3^ mol per tablet) (Sigma Aldrich) and subsequently ground to a very fine powder, over a period of 25 min, with an agate mortar in a dry argon atmosphere. A tablet with a diameter of 10 mm was prepared by placing the finely-ground mixture in between two layers of ~80 mg CaH_2_ and pressing it with a pressure of 3 t for 30–40 s. This tablet was placed in a small tantalum boat inside a nickel tube, which was evacuated to pressures of 10^−3^–10^−5^ mbar. The temperature was gradually increased to 800–1000 °C over a period of 60–120 min and maintained at this level for about 30 min. After cooling to room temperature, the reduction products were retrieved and the metallic ^46^Ti isolated from the co-produced calcium oxide using dilute acetic acid. Further details on the isolation procedure can be found elsewhere (Lommel et al. 2014). The resultant reduced ^46^Ti metal was directly used for the preparation of the targets.

The reduction yield was determined by boiling an aliquot of the reduced product (approx. 5–10 mg) in 2–3 mL concentrated HCl for 10–15 min. Under these conditions, the reduced ^46^Ti metal was dissolved completely (Straumanis and Chen 1951) while the insoluble residue, consisting of TiO and TiO_2_ (Perry 2011, Rumble 2018), was collected and weighed. The ratio of soluble to insoluble species served as an indication for the degree of reduction. X-ray diffraction (XRD) analysis (Philips X’PertPro X-ray diffractometer, wavelength: Cu Kα = 1.541 Å) was additionally employed to identify the chemical speciation of the product.

### Manufacturing and irradiation of ^46^Ti and ^43^CaCO_3_ targets

Targets were prepared by placing 9–28 mg reduced ^46^Ti metal powder or 8–12 mg enriched ^43^CaCO_3_ on top of ~150 mg graphite powder and pressed into pills with a pressure of 5–7 t. The resulting thickness of the target in question was between 0.4 and 0.5 mm with a diameter of 16 mm. After encapsulation in aluminum, the target was introduced into a target holder system. The Injector 2 cyclotron at PSI produces 72 MeV protons and was used for the irradiations presented herein. The required lower beam energies were achieved using niobium degrader discs of various thicknesses (3.2–3.5 mm). ^46^Ti targets were irradiated with proton energies of 15.1 ± 1.9 MeV at beam currents of 30 μA for 60–420 min, whereas, proton energies of 12.0 ± 2.3 MeV and 10.4 ± 2.6 MeV, respectively, at 50 μA were applied to the ^43^CaCO_3_ targets for 90–220 min. The impinging energies were calculated with SRIM-2010 (Ziegler et al. 2010). After the end of bombardment (EOB), the activated targets were detached from the target station and the aluminum encapsulation removed.

### Separation of ^43^Sc from ^46^Ti

**Fig. 2:**
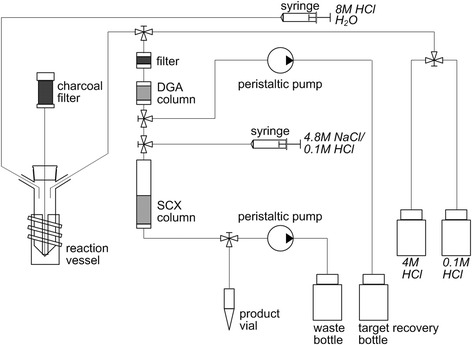
Schematic diagram of the ^46^Ti/^43^Sc separation system

A chemical separation system (schematic shown in Fig. 2) was developed to separate ^43^Sc from ^46^Ti. The irradiated target was transferred into a conical glass vial (30 mL, Schmizo AG, Switzerland) with an integrated charcoal filter (2 mL chromatography column, BioRad, France, filled with charcoal, Merck, Germany)on top. The target was dissolved in 4–5 mL of boiling 8.0 M HCl for 15–20 min. The concentration of the obtained HCl solution was adjusted to ~4.0 M with the addition of MilliQ water. The insoluble graphite disc remained in the glass vial while the radioactive solution was pumped through a filter (1 mL cartridge fitted with a 10 μm frit, ISOLUTE SPE Accessories, UK) before loading onto a 1 mL column cartridge, containing ~85 mg DGA extraction chromatographic resin. Negligible sorption of Ti(III) and a simultaneous strong Sc(III) retention on DGA resin at HCl molarities below 6 M (Pourmand and Dauphas 2010) facilitated its application for the separation of these elements. The reaction vessel was rinsed with 2.0 mL 4.0 M HCl and the solution pumped through the DGA resin. Further 5.0 mL 4.0 M HCl were applied directly onto the DGA column to ensure complete removal of residual ^46^Ti. The ^43^Sc fraction was finally eluted with 4.0 mL 0.1 M HCl directly onto a second column containing SCX cation exchange resin to concentrate the ^43^Sc in a small volume. From this resin, ^43^Sc was eluted with 700 μL 4.8 M NaCl/0.13 M HCl (pH 0–0.5) and partitioned into three fractions. The volumes of the fractions were 100, 400 and 200 μL, respectively, with the second fraction directly used for radiolabeling.

### Separation of ^43^Sc from ^43^Ca

The chemical separation of ^43^Sc from ^43^Ca was performed as for the previously reported procedure for ^44^Sc (van der Meulen et al. 2015). In brief, the irradiated target was dissolved in HCl and the separation of ^43^Sc performed on a DGA extraction chromatographic column. ^43^Sc was directly eluted onto a SCX cation exchange resin cartridge, from where it was eluted in a small volume of NaCl/HCl and directly used for radiolabeling.

### Target recycling

The Ti-containing eluate collected from the DGA column was heated to boiling and the pH adjusted to 8.0 with 25% ammonia solution. As a consequence, a black, flaky precipitate formed immediately and transformed into white TiO_2_ over a waiting period of 40 min. The precipitate was filtered through a glass filter crucible (30 mL, pore size 10–16 μm, Duran Group GmbH, Germany), heated to 400 °C, in air, and kept at this temperature for 1 h to ensure complete oxidation. XRD-measurements were employed for the specification analysis of the recovered target material.

Recycling of the valuable enriched ^43^Ca target material from the collected waste fraction was performed as described previously for ^44^Ca (van der Meulen et al. 2015).

### Radionuclidic purity

A N-type high-purity germanium (HPGe) coaxial detector (Canberra, France) in combination with the InterWinner software package (version 7.1, Itech Instruments, France) was employed for analyzing the radionuclidic purity of the final products. The measurements were performed with an aliquot of 2.5–5.0 MBq ^43^Sc and the counting time adjusted to ensure a statistical measurement error of <5%. Further γ-spectroscopy measurements of the same samples with long counting times were performed several days post-separation, aiming to determine low-level activities originating from long-lived radionuclidic impurities.

### PET phantom study using ^43^Sc

An aliquot was withdrawn from the second fraction of the respective eluate and diluted with 70% ethanol to a volume ratio of 3:1 (aliquot of ^43^Sc eluate: ethanol). The holes (diameter ranging from 0.8–1.3 mm in 0.1 mm steps) of a polycarbonate Derenzo phantom (D = 19.5 mm, H = 15.0 mm) were filled with 600 μL of the diluted solution. Particular care was taken to avoid the inclusion of air bubbles (Bunka et al. 2016). The phantom was closed with a screw cap and aligned on a small-animal PET/CT scanner (eXplore VISTA PET/CT, GE Healthcare, Spain). The determined total radioactivity in the phantom was ~4–8 MBq at the beginning of the PET scan. The energy window was set to 400–700 keV and each phantom scanned for 30 min. Using the post-processing software VivoQuant™ (version 2.00, inviCRO Imaging Service and Software, Boston USA), one representative single transversal section was selected and analyzed at three different phantoms depths. The resulting intensity plots of the profiles were transferred to Origin® 2016 (OriginLab). The full-width at half-maximum (FWHM) was determined for each slice and used for calculating the arithmetic mean and standard deviation. A detailed description of the quantification of the relative resolution is described by Bunka et al. (Bunka et al. 2016).

### Radiolabeling for quality control of the produced ^43^Sc

Radiolabeling of a DOTA-functionalized peptide at pre-defined specific activities was employed for quality control of the product. As non-radioactive metal impurities would compete with the radionuclide for complexation by a DOTA chelator, this method serves as a reasonable benchmark to evaluate the success of a chemical separation (Severin et al. 2012, Valdovinos et al. 2014). After separation of ^43^Sc from the ^46^Ti and ^43^CaCO_3_ target material, the activity of the obtained eluate was quantitatively determined with a dose calibrator (ISOMED 2010, Nuclear-Medizintechnik Dresden, GmbH, Germany – calibrated on a fortnightly basis). The quality of the ^43^Sc was investigated by means of its radiolabeling capability with DOTANOC. The required activity (20–50 MBq) was withdrawn from the vial and mixed with sodium acetate solution (0.5 M, pH 8), in order to obtain a pH of 3.5–4.5, followed by the successive addition of DOTANOC (3.5–14.3 μL of a 0.7 mM solution in MilliQ water, ABX GmbH, Advanced Biochemical Compounds, Germany). The reaction mixture was incubated at 95 °C for 15 min. High performance liquid chromatography (HPLC) with a C-18 reversed-phase column (Xterra™ MS, C18, 5 μm, 150 × 4.6 mm, Waters, USA) was employed in order to determine the radiolabeling yield. Before analysis, the addition of 10 μL 2 mM Na-DTPA solution ensured the complexation of free radiometals. A UV (LaChrom L-7400) and a radiodetector (Berthold, HPLC Radioactivity Monitor, LB 506B) were used for detection. The analysis sequence comprised the gradual change of the mobile phase from 95% A (MilliQ water containing 0.1% trifluoracetic acid) and 5% B (acetonitrile) to 20% A and 80% B, over a period of 15 min and at a flow rate of 1.0 mL/min.

## Results

### Reduction of enriched ^46^TiO_2_

The yield of the reduction procedure of ^46^TiO_2_ to metallic ^46^Ti, performed at GSI, was determined by dissolution of the metallic product in conc. HCl as 95.7%. Comparable yields of 90–98% were verified by the authors of the study when reducing ^50^TiO_2_ and employing energy-dispersive X-ray spectroscopy (EDX) for analysis (Lommel et al. 2014).

While the method using the vacuum-based system at PSI was successful, several parameters were optimized using ^nat^TiO_2_, aiming to enhance the yield of the reduction process. The use of four-fold surplus of reducing agent in a finer granulated form with ^46^TiO_2_ and ^nat^TiO_2_ was found to be a suitable starting mixture. The mixed powder was embedded in between two layers of CaH_2._ Reduction yields of 96–99%, determined by the dissolution test in boiling HCl and verified by XRD analysis, were achieved at pressures <10^−4^ mbar and by increasing the temperature to 950–1000 °C. The XRD spectra are given in the Additional file 1: Figure S2 a-b.

### Production of ^43^Sc from ^46^Ti via the (p,α) nuclear reaction

The activities of the ^46^Ti-targets at the end of bombardment (EOB) ranged between 60 and 225 MBq ^43^Sc, however, theoretically achievable activities (A(^43^Sc)_calc_) were estimated to be between 590 and 2340 MBq (Table 1). The calculations were performed by taking into account the mass of ^46^Ti, the irradiation time (t_irr_), the proton flux (Φ) and the cross section for the reaction ^46^Ti(p,α)^43^Sc amounting to 36 ± 2 mbarn (Carzaniga et al. 2017). Differences between the experimental and the calculated activity are expressed by the factor f(^43^Sc), which were typically in the range between 5.6 and 12.3. The values of f(^43^Sc) characterize how many times larger the theoretical activity is than that experimentally obtained. In one particular production run, an exceptionally high activity of ~1.0 GBq ^43^Sc was generated under identical irradiation conditions. The resulting, rather low value of f(^43^Sc) = 1.9, clearly demonstrates the potential of this approach. Formulae used for the calculations of A(^43^Sc)_calc_ and f(^43^Sc) are given in the Additional file 1: Figure S1 a-b.

**Table 1:** Comparison between the experimental A(^43^Sc)_exp_ and the calculated activities A(^43^Sc)_calc_, obtained from proton irradiations of enriched ^46^Ti targets, measured at EOB

Number of irradiations	m(^46^Ti) [mg]	t_irr_ [min]	A(^43^Sc)_exp_ at EOB [MBq]	A(^43^Sc)_calc_ [MBq]	f(^43^Sc)
2	10	180–240	110–140	930–990	7.2–8.4
5	11	110–240	60–180	590–1080	6.0–12.2
3	12	120–240	130–150	700–1180	5.6–8.7
1	15	390	1030	1990	1.9
1	16	420	225	2210	9.9
1	17	420	190	2340	12.3

### Chemical behavior of ^43^Sc and ^46^Ti on DGA resin and their separation

**Fig. 3:**
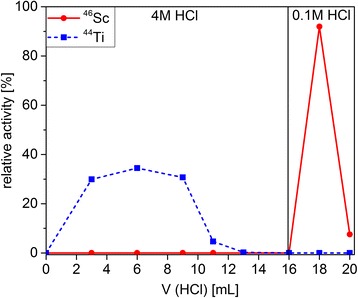
Elution profile of ^44^Ti/^nat.^Ti (blue squares) and ^46^Sc (red dots) on DGA extraction chromatographic resin. Each fraction was measured by γ-spectroscopy until the statistical measurement error was <5%

The chemical behavior of Sc(III) and Ti(III) on DGA resin was investigated using ~30 mg naturalTi metal, spiked with trace amounts of radioactive ^46^Sc (1.7 kBq) and ^44^Ti (2.3 kBq). γ-Spectroscopy was employed in order to quantify the ^46^Sc and ^44^Ti radioactivity in each fraction (the resulting elution profile is shown in Fig. 3). Using 9.2 mL 4.0 M HCl solution, Sc(III) was quantitatively sorbed on DGA resin, while Ti(III) was not retained. Before the final elution of Sc(III) with 4.0 mL 0.1 M HCl, the resin was rinsed with additional 5.0 mL 4.0 M HCl to ensure complete removal of Ti(III).

With the developed separation system (Fig. 2), 89.7 ± 3.1% of the total ^43^Sc activity could be eluted in a small volume using 4.8 M NaCl/0.13 M HCl as eluent. Fractionized collection revealed that ~90% of the eluted ^43^Sc activity were obtained in the second fraction (400 μL), the remaining 10% were divided between the first (100 μL) and third fraction (200 μL). The residual overall ^43^Sc activity (~10%) was left on the graphite disc, the DGA and SCX resin columns, respectively.

### Production of ^43^Sc from ^43^Ca via the (p,n) nuclear reaction and separation

Total activities of 380–720 MBq were generated by the irradiation of ^43^CaCO_3_ targets, which consisted of 66.2% ^43^Sc and 33.3% co-produced ^44^Sc (Table 3), consequently, resulting in final yields of 250–480 MBq pure ^43^Sc radioactivity at EOB (Table 2). Theoretically accessible activities of 1270–3200 MBq ^43^Sc were calculated based on beam energies of 12 MeV at a corresponding cross section of 275 mbarn (Carzaniga et al. 2017). The discrepancies between the experimental and the calculated activities, once more expressed by the factor f(^43^Sc), were within the range between 3.9 and 6.8, which is slightly lower than those determined for the ^46^Ti(p,α)^43^Sc route.

**Table 2:** Comparison of the measured total activities A(^43/44^Sc)_exp_, the experimental ^43^Sc activities A(^43^Sc)_exp_ as well as the calculated activities A(^43^Sc)_calc_, obtained from proton irradiations of enriched ^43^CaCO_3_ targets, measured at EOB

Number of irradiations	m(^43^CaCO_3_) [mg]	t_irr_ [min]	A(^43/44^Sc)_exp_ at EOB [MBq]	A(^43^Sc)_exp_ at EOB [MBq]	A(^43^Sc)_calc_ [MBq]	f(^43^Sc)
1	8	90	380	250	1270	5.0
4	9	90–220	440–670	290–440	1420–2910	3.9–6.6
1	10	220	720	480	3200	6.8

The isolation of 90.4 ± 5.5% of the total ^43^Sc/^44^Sc activity was possible in a small volume (700 μL) of 4.8 M NaCl/0.13 M HCl eluent by using the previously-developed separation system (van der Meulen et al. 2015). The residual ~10% of ^43^Sc/^44^Sc activity were left on the remaining components of the setup, e.g. graphite disc, DGA and SCX resin columns, respectively.

### Target recycling

A γ-spectroscopic measurement of the ^46^Ti-containing fraction indicated the presence of ^48^V (T_1/2_ = 16 days), presumably being formed in the nuclear reaction ^48^Ti(p,n)^48^V. In order to avoid any co-precipitation, the liquid was set aside until ^48^V was completely decayed to stable ^48^Ti. Consequently, the target recycling process was developed with natural titanium. The achieved overall recovery yield for the precipitation of ^nat^TiO_2_ was 97.6%, with XRD measurements confirming the chemical identity of the product. The XRD spectrum is given in the Additional file 1: Figure S3.

The recovery of enriched ^43^Ca target material was performed according to the previously-described method used for ^44^Ca (van der Meulen et al. 2015) at an equivalent efficiency. The ^43^Sc obtained by irradiation of recovered material, proved to be of the same quality as with targets from newly-purchased ^43^CaCO_3_. An unchanged radionuclidic purity of the obtained ^43^Sc eluate confirmed the absence of trace element impurities in the recycled ^43^CaCO_3_.

### Radionuclidic purity of ^43^Sc produced from ^46^Ti and ^43^Ca target material

Irradiation of ^46^Ti targets (97.0% enriched) with protons yielded a product of high radionuclidic purity, containing 98.2% ^43^Sc and only 1.5% ^44^Sc. In comparison, the ^43^Sc eluate isolated from proton irradiated ^43^CaCO_3_ (57.9% enriched) contained 66.2% ^43^Sc and 33.3% ^44^Sc. Long-term γ-spectroscopic measurements determined low activity levels of 0.079% ^44m^Sc, ^46^Sc, ^47^Sc, ^48^Sc and 0.34% ^44m^Sc, ^47^Sc, ^48^Sc in the final products of irradiated ^46^Ti and ^43^Ca targets, respectively (Table 3). All radionuclides of Sc were formed in (p,n), (p,2n), (p,α) and (p,2p) nuclear reactions, with stable isotopes of titanium (^46^Ti, ^47^Ti, ^48^Ti, ^49^Ti, ^50^Ti) and calcium (^40^Ca, ^42^Ca, ^43^Ca, ^44^Ca, ^46^Ca, ^48^Ca), being present in the respective target material. In the case of the ^46^Ti/^43^Sc production route, the amount of ^46^Sc was found to exceed the prediction by a factor of ~50, while no long-lived ^46^Sc, even if predicted, could be determined in the final eluate available from the ^43^Ca/^43^Sc route. Low levels of various Y radionuclides (^86^Y, ^87^Y, ^87m^Y and ^88^Y) were present in products isolated from ^46^Ti and ^43^CaCO_3_ target material, in total, amounts of 0.29% and 0.19%, respectively. No Y isotopes were observed in the ^43^Sc eluate obtained from ^46^Ti target material which was reduced at PSI, however.

**Table 3:** The radionuclide inventory of the ^43^Sc eluate, isolated from irradiated ^46^Ti and ^43^CaCO_3_ targets is shown, together with the calculated values at EOB. Cross section data for the nuclear reactions ^46^Ti(p,α)^43^Sc, ^43^Ca(p,n)^43^Sc and ^44^Ca(p,n)^44^Sc were taken from Carzaniga et al. (Carzaniga et al. 2017), while the data for all other nuclear reactions was retrieved from the TENDL 2015 database (Koning et al. 2015)

^43^Sc eluate isolated from irradiated ^46^Ti				
		Radionuclide inventory at EOB [%]		
Isotope	Nuclear reaction	Calculated prediction	Experimental results	
		14.6/15 MeV	15.1 ± 1.9 MeV	
^43^Sc	^46^Ti(p,α)^43^Sc	99.1	98.2 ± 0.3	
^44g^Sc	^47^Ti(p,α)^44g^Sc	0.9	1.5 ± 0.2	
^44m^Sc	^47^Ti(p,α)^44m^Sc	1.3 × 10^−2^	4.2 × 10^−2^ ± 1.6 × 10^−2^	
^46^Sc	^47^Ti(p,2p)^46^Sc^49^Ti(p,α)^46^Sc	2.2 × 10^−4^	1.1 × 10^−2^ ± 5.7 × 10^−3^	
^47^Sc	^50^Ti(p,α)^47^Sc^48^Ti(p,2p)^47^Sc	3.0 × 10^−3^	9.6 × 10^−3^ ± 4.7 × 10^−3^	
^48^Sc	^49^Ti(p,2p)^48^Sc	1.6 × 10^−7^	1.7 × 10^−2^ ± 7.0 × 10^−3^	
^86^Y, ^87^Y, ^87m^Y, ^88^Y		^–^	0.16, 2.7 × 10^−2^, 9.5 × 10^−2^, 1.0 × 10^−2^	
^43^Sc eluate isolated from irradiated ^43^CaCO_3_				
		Radionuclide inventory at EOB [%]		
Isotope	Nuclear reaction	Calculated prediction		Experimental results
		9.9/10.0 MeV	12.4/12.0 MeV	10.4 ± 2.6/12.1 ± 2.3 MeV
^43^Sc	^43^Ca(p,n)^43^Sc	67.4	65.5	66.2 ± 1.5
^44g^Sc	^44^Ca(p,n)^44g^Sc	32.4	34.3	33.3 ± 1.5
^44m^Sc	^44^Ca(p,n)^44m^Sc	0.1	0.2	0.2 ± 5.3 × 10^−2^
^46^Sc	^46^Ca(p,n)^46^Sc	1.5 × 10^−5^	1.2 × 10^−5^	–
^47^Sc	^48^Ca(p,2n)^47^Sc	2.0 × 10^−2^	2.6 × 10^−2^	2.2 × 10^−2^ ± 1.0 × 10^−2^
^48^Sc	^48^Ca(p,n)^48^Sc	5.7 × 10^−2^	2.3 × 10^−2^	0.1 ± 2.9 × 10^−2^
^86^Y, ^87^Y		–	–	0.2, 1.0 × 10^−2^

### PET phantom study

**Fig. 4:**
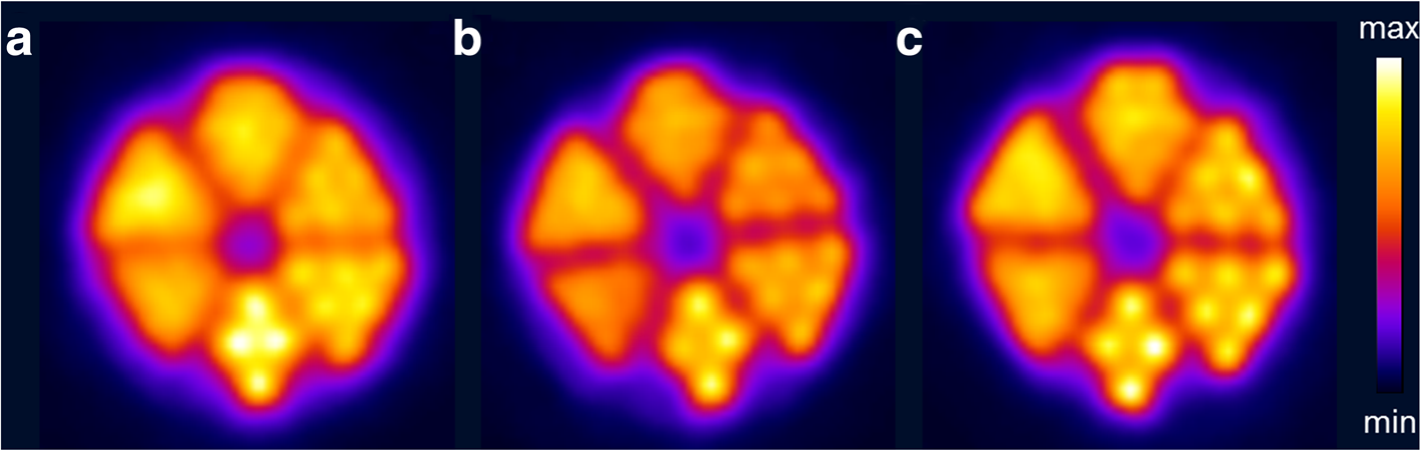
Transversal slices of PET scans of Derenzo phantoms (hole diameter ranging from 0.8–1.3 mm in 0.1 mm steps) filled with >99% ^44^Sc (**a**), 66.2% ^43^Sc (**b**) and 98.2% ^43^Sc (**c**). The acquisition of the PET scans was performed in an energy window of 400–700 keV for 30 min, in order to obtain a total number of ~6 × 10^7^ coincidences

PET images of Derenzo phantoms were acquired on a small-animal PET/CT scanner using ^44^Sc as well as ^43^Sc, produced via the ^46^Ti and ^43^Ca routes (radionuclidic purity: 98.2% and 66.2% ^43^Sc), respectively. A simple visual comparison already suggests a favorable image quality and an improved resolution for ^43^Sc in comparison to ^44^Sc (Fig. 4). The numerical expression of these differences was derived by means of the FWHM, which was determined for a hole diameter of 1.3 mm. The resolution was found to be the best for ^43^Sc obtained from ^46^Ti, followed by ^43^Sc from ^43^Ca and, finally, by ^44^Sc (Table 4), hence, the calculated FWHM values corroborate the visual evaluation. The observed sequence is in agreement with the expectations according to the average positron energies of the respective radionuclides.

**Table 4:** FWHM determined for phantom hole-diameters of 1.3 mm for ^44^Sc and ^43^Sc in three different sections of the PET scan

Radionuclide	Radionuclidic purity [%]	Eβ^+^_average_ [keV]	FWHM [mm]
^44^Sc	>99 (<1% ^44m^Sc)	632	2.12 ± 0.04
^43^Sc from ^43^Ca	66.2 (33.3% ^44^Sc)	476	2.04 ± 0.06
^43^Sc from ^46^Ti	98.2 (1.5% ^44^Sc)	476	1.87 ± 0.14

### Quality control of ^43^Sc

**Fig. 5:**
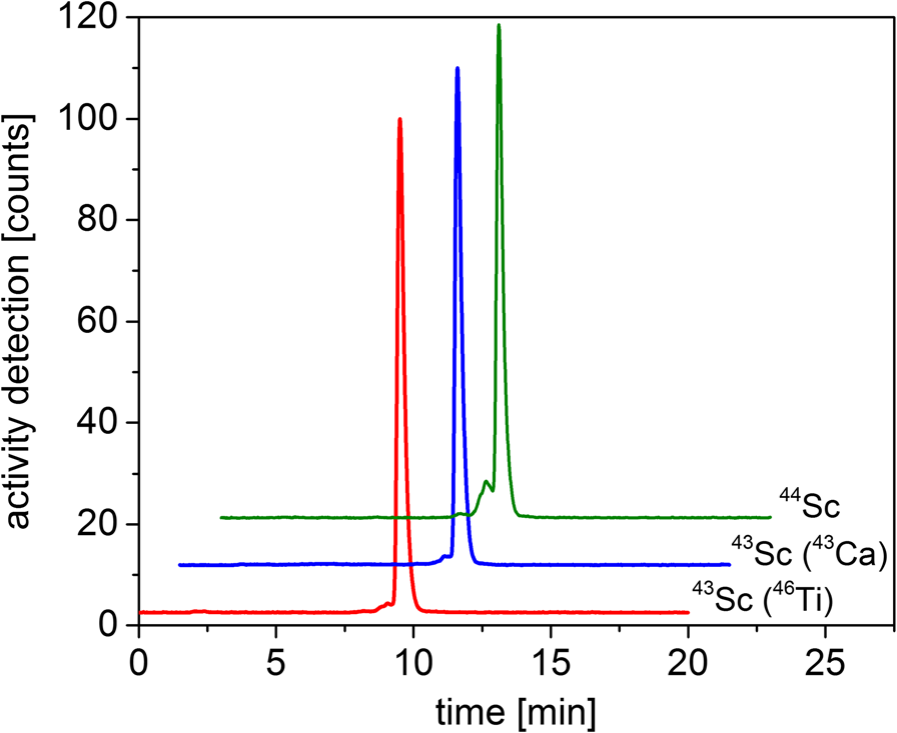
HPLC chromatograms of ^43^Sc isolated from ^46^Ti and ^43^CaCO_3_ target material and ^44^Sc, directly after the radiolabeling reaction with DOTANOC (the chromatograms of ^43^Sc- (^43^Ca) and ^44^Sc-DOTANOC are shifted up and sideways for better visibility). The retention times of free ^43^Sc and ^44^Sc were determined to be 2.2 ± 0.2 min and 9.7 ± 0.3 min for ^43^Sc/^44^Sc-DOTANOC

Quality control of the ^43^Sc (Fig. 5) was performed by radiolabeling of DOTANOC, isolated from irradiated ^43^CaCO_3_ targets. Reproducibly, >96% radiochemical purity were achieved at a specific activity of 8 MBq ^43^Sc per nmol DOTANOC. Lower activity concentrations of the ^43^Sc eluate, obtained from irradiated ^46^Ti targets, rendered radiolabeling at high specific activities more challenging. The radiolabeling reaction could be reproducibly performed at 5 MBq/nmol.

## Discussion

Enriched ^46^Ti is only commercially available as ^46^TiO_2_. As titanium dioxide requires hot sulfuric or hydrofluoric acid for dissolution, the reduction prior to target manufacturing was necessary. After conversion to the metallic state, the dissolution of ^46^Ti under less stringent conditions becomes possible. The initial reduction process described elsewhere (Lommel et al. 2014) was changed to a vacuum-based system. A four-fold molar excess of reducing agent in a fine granulated formulation with ^46^TiO_2_, pressures <5 × 10^−3^ mbar and temperatures of 950–1000 °C were identified to be crucial conditions to achieve maximum yields. The optimized procedure allowed for reduction yields between 96% and 99%, determined by the dissolution test in concentrated HCl and verified by XRD analysis (Additional file 1: Figure S2 a-b).

Activities of 60–225 MBq ^43^Sc could be regularly obtained by the irradiation of the enriched ^46^Ti targets, while 250–480 MBq ^43^Sc (equivalent to total activities of 380–720 MBq ^43^Sc/^44^Sc) was produced via the ^43^Ca(p,n)^43^Sc nuclear reaction. The 2–4-fold higher ^43^Sc activity available from ^43^Ca irradiations can be attributed to the higher nuclear cross section of the ^43^Ca(p,n)^43^Sc reaction in comparison to the ^46^Ti(p,α)^43^Sc nuclear reaction (Additional file 1: Figure S4). A theoretical ^43^Sc yield obtainable via the ^46^Ti route, was calculated to be 0.6–2.3 GBq, whereas 1.3–3.2 GBq ^43^Sc were calculated for the ^43^Ca route. The discrepancies to the experiment results in this work can be mainly explained by the rather large energy degradation of the proton beam (using niobium), from the initial 72 MeV to ~10–15 MeV, resulting in a beam with a broad spread of energies at diminished intensities. Another addition to the discrepancy of results is the lack of beam diagnostics closer than 80 cm from the target. The exceptionally high yield of ~1 GBq became attainable when the proton beam was precisely positioned, at a specific energy, on the target material.

A product of high radionuclidic purity was isolated from irradiated ^46^Ti samples, containing only 1.5% ^44^Sc and 0.079% of all other Sc radioisotopes, which is comparable to the calculations determined from the cross section measurements (Koning et al. 2015, Carzaniga et al. 2017). The quantity of ^46^Sc (0.011%, T_1/2_ = 83.8 d) was about 50 times higher than expected, however, the percentage can be still related to the threshold value for the long-lived ^68^Ge (T_1/2_ = 270.8 d) impurity in the ^68^Ga generator eluate (0.001%) set by the European Pharmacopoeia (Council of Europe 2013). γ-Spectroscopic measurements of the ^43^Sc eluate, isolated from irradiated ^43^CaCO_3_ targets, revealed the presence of 66.2% ^43^Sc and 33.3% ^44^Sc. The co-formation of ^44^Sc originated from ^44^Ca (12.36% in the target material) via the (p,n) nuclear reaction, as the cross section maxima of both reactions, ^44^Ca(p,n)^44^Sc and ^43^Ca(p,n)^43^Sc, are centered around 9–10 MeV (Additional file 1: Figure S4). Performing the irradiation with proton energies of 10 or 12 MeV did not influence the ratio of ^43^Sc and ^44^Sc, which is in compliance with the calculations performed (Koning et al. 2015, Carzaniga et al. 2017), hence, the isolated product can be rather considered as a combination of two PET nuclides, with comparable decay properties. Trace activities of various Y radioisotopes were identified in both products, conceivably produced via (p,n) and (p,α) nuclear reactions with ^nat^Sr and ^nat^Zr impurities, both being present as low-level contaminants in the target materials (Additional file 1: Table S5). A scan for impurities of trace metals by ICP-OES in enriched ^46^TiO_2_ and ^46^Ti metal suggests that they were probably introduced in the course of the reduction process performed at GSI. These impurities were later eliminated with the use of calcium hydride of higher chemical purity, hence, the introduction of trace metals was avoided, confirmed by the absence of any Y isotopes in the ^43^Sc eluate. The limited quantity and high costs of ^43^CaCO_3_ did not allow for a similar analysis.

The separation of ^43^Sc from ^43^Ca target material was performed at a comparable yield (90.4 ± 5.5%) to the previous separations of ^44^Sc and ^47^Sc from irradiated ^44^Ca and ^46^Ca targets (98.0 ± 0.3% and 94.8 ± 2.1%) (van der Meulen et al. 2015, Domnanich et al. 2017). Using the developed ^46^Ti/^43^Sc separation system, about 90% of the initial ^43^Sc activity could be isolated within 45 min, which is about 10 times faster compared to all previously-reported radiochemical separation methods in this regard (Kolsky et al. 1998).

Radiolabeling of ^43^Sc with the model compound DOTANOC was utilized to assess the chemical purity of both eluates, obtained by irradiation from ^46^Ti and ^43^CaCO_3_ target material, respectively (Severin et al. 2012, Valdovinos et al. 2014). Reproducible radiosynthesis of ^43^Sc-DOTANOC was demonstrated at specific activities of 5–8 MBq/nmol. These results are slightly lower than previously achieved with eluates of ^44^Sc and ^47^Sc (van der Meulen et al. 2015, Domnanich et al. 2017), but still comparable when taking the obtained low ^43^Sc activity concentrations into consideration.

The establishment of a recovery process for the enriched ^46^Ti target material was an important part of the present study. A simple, fast and efficient method was employed to precipitate TiO_2_ with a high overall yield, however, it was only performed with natural titanium thus far. The established method for ^44^Ca was successfully employed, for the recycling of the enriched ^43^Ca target material. Both recycling methods have great potential to significantly reduce the production cost as the current prices for the target materials amount to about 30 USD and 260 USD for 1 mg of enriched ^46^Ti and ^43^Ca, respectively.

The resolution of a PET image is, among other factors, influenced by the radionuclide’s positron range. The discriminability between two different radioactive sources in an image is described by the spatial resolution which is, on a particular PET system, only dependent on the radionuclide’s positron energy (Palmer et al. 2005). Herein it was shown, that scanning at slightly improved resolution is demonstrated when using ^43^Sc instead of ^44^Sc. An intermediate resolution is shown with ^43^Sc obtained from ^43^Ca (66.2% radionuclidic purity) target material. These findings are in line with the expectations, based on the correlation between lower positron energy and enhanced image quality. To date, no FWHM values have been published for ^43^Sc, but the results in this work are comparable with previously published data for ^11^C and ^89^Zr (Palmer et al. 2005, Bunka et al. 2016). This modest gain in resolution, demonstrated using a small animal PET scanner, is unlikely to play a significant role in clinical practice. Speculation whether the co-emitted γ-radiation of ^43^Sc (Eγ = 372 keV [23%]) will interfere with the image resolution of a clinical PET scanner should not emerge, as modern PET systems based on scintillation crystals use energy windows in between 430 and 650 keV for the detection of annihilation photons (the γ-energy lies outside this range and should be cut off by the standard energy window) (Gnesin 2017). The absence of co-emitted high energetic gamma rays as they are emitted by ^44^Sc at high intensity (Eγ = 1157 keV [100%]), would be clearly advantageous regarding the dose burden to patients and medical staff.

## Conclusion

The production of several hundred MBq ^43^Sc was demonstrated for the first time by proton irradiation of enriched ^46^Ti and ^43^Ca. Only moderate proton energies of 10–15 MeV are required for the respective nuclear reactions, ^46^Ti(p,α)^43^Sc and ^43^Ca(p,n)^43^Sc, which are available from most commercial biomedical cyclotrons. It was shown that higher ^43^Sc radioactivities were produced via the ^43^Ca route. At the same time, this production pathway is accompanied by a comparatively less complex target preparation and separation procedure. Even though the irradiation of ^46^Ti yielded a product of higher radionuclidic purity, the eluate obtained from irradiated ^43^CaCO_3_ can be rather considered as combination of two PET nuclides. The cost of enriched ^43^Ca is significantly higher than that of ^46^Ti, however, the expenses are kept within limits by the implementation of recycling procedures. Based on the results obtained, it can be concluded that the ^43^Ca route features several advantages over the ^46^Ti path when aiming at a production of ^43^Sc on a routine basis. In future, the production will be optimized to reproducibly obtain high quantities of ^43^Sc in order to assess the gain in resolution in a clinical setting. Further studies in this regard are necessary for the upscale of production by means of increasing target material quantities. This is currently underway, utilizing a biomedical cyclotron.

## Additional file

Additional file 1 Supplementary experimental data. (DOCX 445 kb)

## Abbreviations

CT: computed tomography
DOTA: 1,4,7,10-tetraazacyclododecane-1,4,7,10-tetraacetic acid
EOB: end of bombardment
EOS: end of separation
FWHM: full-width at half-maximum
GMP: good manufacturing practice
HPLC: high performance liquid chromatography
p.i.: post injection
PET: positron emission tomography
SPECT: single photon emission computed tomography
