# Supplementary material for: Production and separation of 43Sc for radiopharmaceutical purposes
Source: EJNMMI Radiopharm Chem. 2017 Nov 25;2:14. doi: 10.1186/s41181-017-0033-9 (PMC5824704; doi:10.1186/s41181-017-0033-9)
Supplement: Supplementary file 1 — Supplementary experimental data. (DOCX 445 kb) [file 41181_2017_33_MOESM1_ESM.docx]

Supplementary material

**Production and separation of ^43^Sc for radiopharmaceutical purposes**

Katharina A. Domnanich^1,2^, Robert Eichler^1,2^, Cristina Müller^3,4^, Sara Jordi^1,2^, Vera Yakusheva^5^, Saverio Braccini^6^, Martin Behe^1,3^, Roger Schibli^3,4^, Andreas Türler^2^, Nicholas P. van der Meulen^1,3*^

*^1^Laboratory of Radiochemistry, Paul Scherrer Institute, 5232 Villigen PSI, Switzerland*

*^2^Department of Chemistry and Biochemistry University of Bern, 3012 Bern, Switzerland*

*^3^Center for Radiopharmaceutical Sciences ETH-PSI-USZ, Paul Scherrer Institute, 5232 Villigen PSI, Switzerland*

*^4^Department of Chemistry and Applied Biosciences, ETH Zurich, 8093 Zurich, Switzerland*

*^5^GSI Helmholtzzentrum für Schwerionenforschung GmbH, 64291 Darmstadt, Germany*

*^6^Albert Einstein Center for Fundamental Physics (AEC), Laboratory for High Energy Physics (LHEP), University of Bern, Sidlerstrasse 5, 3012 Bern, Switzerland*

E-mail addresses:

[katharina.domnanich@psi.ch](mailto:katharina.domnanich@psi.ch); [robert.eichler@psi.ch](mailto:robert.eichler@psi.ch), [cristina.mueller@psi.ch](mailto:cristina.mueller@psi.ch); [sara.jordi@psi.ch](mailto:alain.blanc@psi.ch); [v.yakusheva@gsi.de](mailto:v.yakusheva@gsi.de); [saverio.braccini@lhep.unibe.ch](mailto:saverio.braccini@lhep.unibe.ch); [martin.behe@psi.ch](mailto:martin.behe@psi.ch); [roger.schibli@psi.ch](mailto:roger.schibli@psi.ch); [andreas.tuerler@dcb.unibe.ch](mailto:andreas.tuerler@dcb.unibe.ch); [nick.vandermeulen@psi.ch](mailto:nick.vandermeulen@psi.ch)

* Corresponding author:

Dr. Nicholas P. van der Meulen

Laboratory of Radiochemistry

Paul Scherrer Institute

5232 Villigen-PSI

Switzerland

e-mail: nick.vandermeulen@psi.ch

phone: +41-56-310 50 87

fax: +41-56-310 28 49

$${A\left( {}^{43}{Sc} \right)}_{calc}=\sigma*\phi*N_{T}*(1-e^{-\lambda*t_{irr}})$$

**Supplementary Fig. S1a** Formula for the calculation of the ^43^Sc activity in Bq (s^-1^), accessible under the applied irradiation conditions. σ = nuclear cross section of the ^46^Ti(p,α)^43^Sc and ^43^Ca(p,n)^43^Sc reaction in cm^-2^, N_T_ = number of ^46^Ti or ^43^Ca atoms, Φ = proton flux in protons*s^-1^, λ = decay constant of ^43^Sc in s^-1^, t_irr_ = irradiation time in s

$$f({}^{43}{Sc)}=\frac{{A({}^{43}{Sc)}}_{calc}}{{A({}^{43}{Sc)}}_{exp}}$$

**Supplementary Fig. S1b** Formula for the discrepancy $f$ between the theoretically achievable and the experimentally obtained ^43^Sc activity (dimensionless).


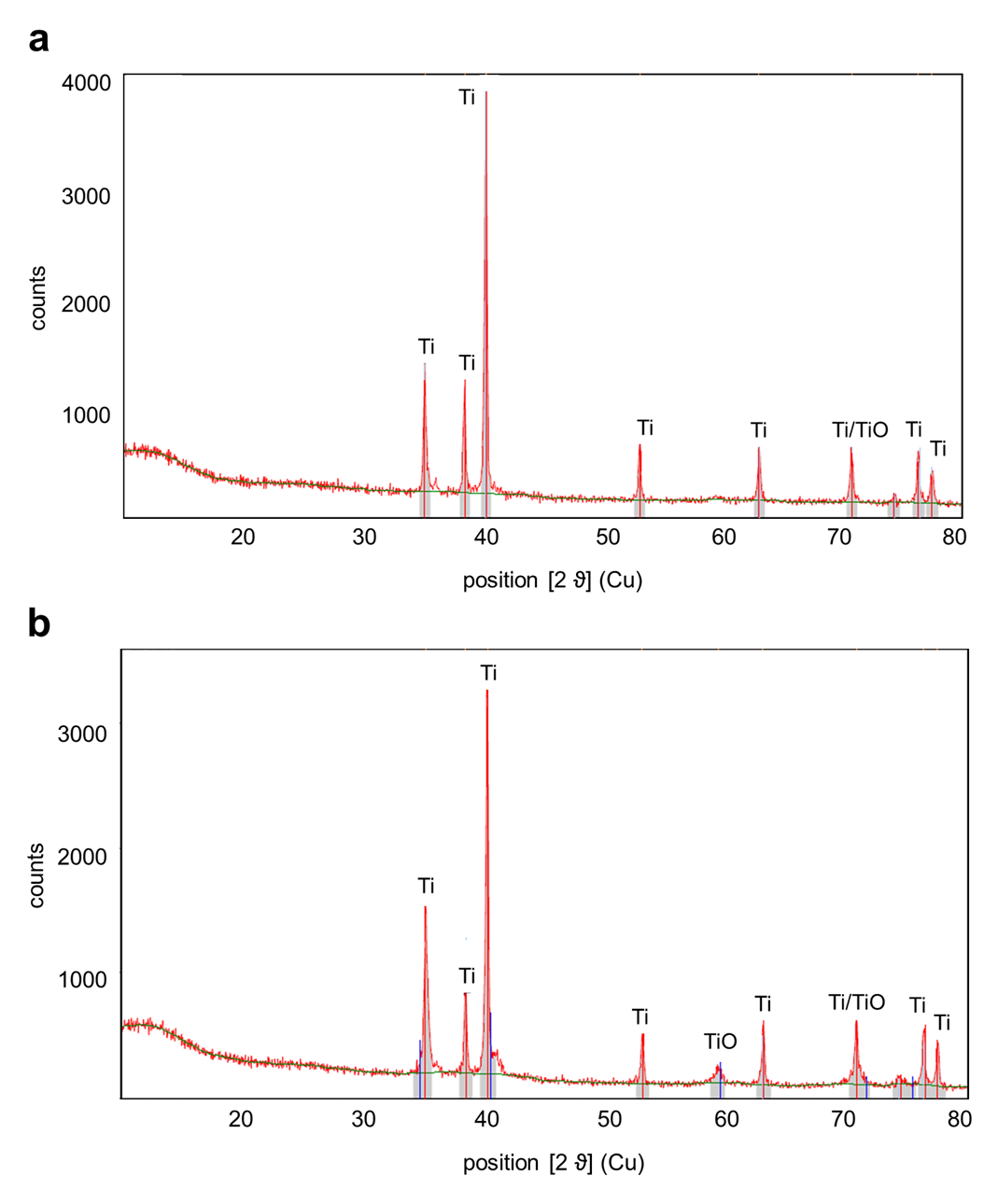


**Supplementary Fig. S2** XRD Spectra of reduced ^46^Ti metal of a sample with a determined reduction yield of 99% (**a**) and 96% (**b**). The remaining species, insoluble by the HCl dissolution, were identified as TiO in both cases.


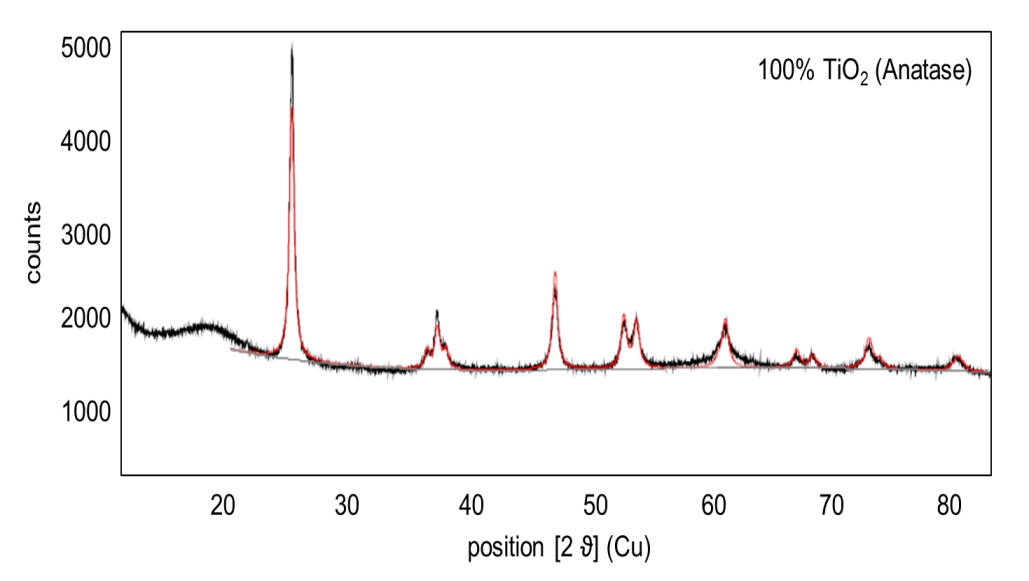


**Supplementary Fig. S3** XRD Spectrum of precipitated, natural TiO_2_ after drying at 400 °C for 1 h.


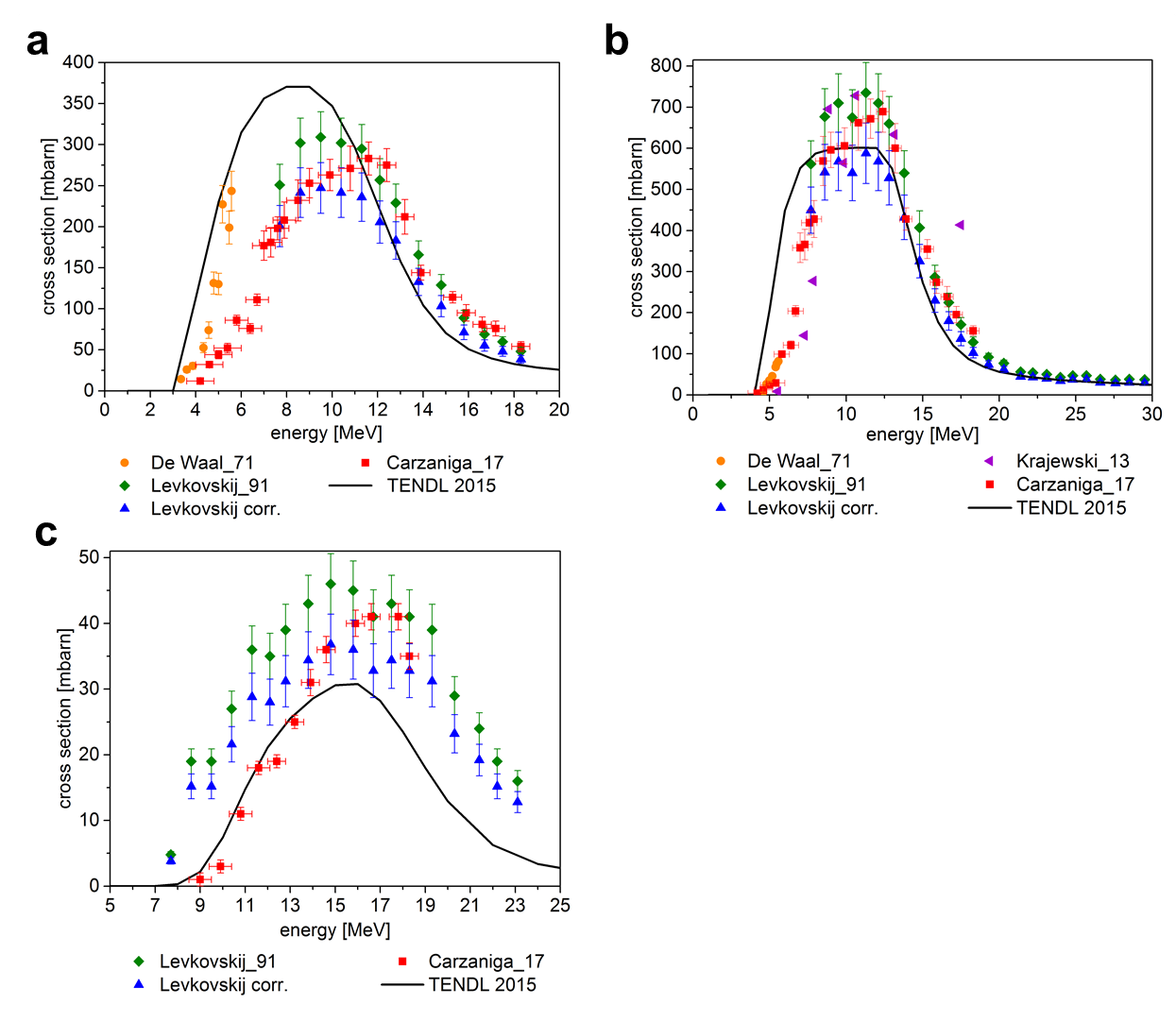


**Supplementary Fig. S4** Measured cross section values (squares, triangles and dots, retrieved from the EXFOR database and Carzaniga et al.) (Carzaniga, Auger et al. 2017, International Atomic Energy Agency 2017) as well as the theoretical calculations from the TENDL-2015 library (Koning, Rochman et al. 2015) for the ^43^Ca(p,n)^43^Sc (**a**), ^44^Ca(p,n)^44^Sc (**b**) and the ^46^Ti(p,α)^43^Sc (**c**) nuclear reaction

**Supplementary Table S5** Trace metal analysis of the purchased ^46^TiO_2_ and reduced ^46^Ti metal by ICP-OES. Only the elements determined at a concentration higher than the detection limit are listed below

| Element | Ratio of element per mg of Ti | |
| --- | --- | --- |
|  | purchased ^46^TiO_2_ | reduced to ^46^Ti metal |
| Ca | 3.7*10^-5^ | 4.9*10^-3^ |
| Mg | 5.2*10^-6^ | 1.8*10^-4^ |
| Sr | 2.4*10^-7^ | 1.1*10^-3^ |
| Ti | 1 | 1 |
| Zr | 3.4*10^-5^ | 6.6*10^-4^ |

**References**

Carzaniga, T. S., M. Auger, S. Braccini, M. Bunka, A. Ereditato, K. P. Nesteruk, P. Scampoli, A. Türler and N. Van der Meulen (2017). "Measurement of ^43^Sc and ^44^Sc production cross-section with an 18 MeV medical PET cyclotron." Appl Radiat Isot.

International Atomic Energy Agency. (2017). "Experimental Nuclear Reaction Data (EXFOR)." Retrieved 04.09.2017, from https://www-nds.iaea.org/exfor/exfor.htm.

Koning, A. J., D. Rochman, J. Kopecky, C. Sublet, E. Bauge, S. Hilaire, P. Romain, B. Morillon, H. Duarte, S. van der Marck, S. Pomp, H. Sjostrand, R. Forrest, H. Henriksson, O. Cabellos, S. Goriely, J. Leppanen, H. Leeb, A. Plompen and R. Mills. (2015). "TENDL-2015: TALYS-based evaluated nuclear data library." Retrieved 05.03.2017, from https://tendl.web.psi.ch/tendl_2015/tendl2015.html.
